# Editorial: Cytokine-Ion Channel Interactions in Pulmonary Inflammation

**DOI:** 10.3389/fimmu.2018.02598

**Published:** 2018-11-06

**Authors:** István Vadász, Rudolf Lucas

**Affiliations:** ^1^Department of Internal Medicine, Justus Liebig University, Universities of Giessen and Marburg Lung Center, Member of the German Center for Lung Research, Giessen, Germany; ^2^Vascular Biology Center, Medical College of Georgia, Augusta University, Augusta, GA, United States; ^3^Department of Pharmacology and Toxicology, Medical College of Georgia, Augusta University, Augusta, GA, United States; ^4^Division of Pulmonary and Critical Care Medicine, Medical College of Georgia, Augusta University, Augusta, GA, United States

**Keywords:** ion channel, pulmonary inflammation, edema, cytokines, alveolar liquid clearance rate

The remarkable architecture of our airways, with 23 levels of branching from the trachea to the approximately 600 million alveoli in an adult lung, accounts for an alveolar epithelial exchange surface of up to 100 m^2^; several 100-fold higher than the surface area of a sphere with a similar volume. These alveoli, together with the surrounding capillaries, form the air-blood barrier, the primary site of gas exchange, allowing O_2_ uptake from the alveolar space and CO_2_ removal from the blood (1). This complex, but vulnerable system requires that alveoli remain relatively dry and that alveolar-capillary barriers are tight, as assured by tight and adherens junction proteins (2). Ion channels and transporters are crucially involved in keeping the alveolar space dry by promoting alveolar liquid clearance (ALC). Vectorial sodium transport through the apically expressed epithelial sodium channel (ENaC) and the basolateral sodium-potassium pump (Na^+^,K^+^-ATPase) in type II and I alveolar epithelial cells mediates reabsorption of liquid from the alveolar into the interstitial space, from where it is removed by the lymphatic system Hamacher et al.; Huppert and Matthay; Wynne et al.; Peteranderl et al.; Vadász and Sznajder. Unfortunately, under clinical conditions such as acute lung injury (ALI), ARDS or severe pneumonia, vectorial Na^+^ transport is often impaired (3).

In this Research Topic contributed by an international consortium of researchers, several novel aspects of cytokine-ion transporter interactions are reported in the context of lung inflammation and injury. Wynne et al. review how the disturbed balance between pro- and anti-inflammatory cytokines (such as TNF-α, TGF-β1, IL-1β and IFN-γ, and IL-10 and IL-1 receptor antagonists) can shift the system to impaired ALC and to development of pulmonary edema. The authors focus on mechanisms by which pro-inflammatory cytokines affect surface expression, maturation and open probability of ENaC. Apart from impaired ALC, also compromised alveolar-capillary barriers can accompany ARDS, ALI and severe pneumonia. Hamacher et al. provide an overview of recent literature on how inflammation depends on complex and time-dependent co-signaling, interactions between the involved cell types, as well as on cell demise and barrier dysfunction. They reflect on how fluid reabsorption can still function in the presence of impaired alveolar epithelial-capillary barriers and reduced expression of ion transporters, with a special emphasis on ischemia-reperfusion lung injury upon lung transplantation.

Because the alveolar-epithelial barrier is an integral component of the mammalian innate immune system, the effects of bacterial and viral infections of the distal airways on Na^+^ transporters, ALC and integrity of the barrier itself are of high pathophysiological and clinical relevance. In a series of *in vivo* and *in vitro* studies, Brazee et al. demonstrate a central role for the pro-inflammatory protein FXYD5, a tissue-specific regulator of the Na^+^,K^+^-ATPase expression, which is upregulated in alveolar epithelial cells upon Gram-negative infection, in alveolar barrier damage secondary to lipopolysaccharide treatment. The authors establish that this detrimental effect is primarily mediated by NF-κB-dependent epithelial production of C-C chemokine ligand-2 (CCL2) and subsequent recruitment of monocytes to the lung. A better understanding of the mechanisms by which alveolar epithelial FXYD5 modulates cytokine and chemokine expression may foster the development of new therapies for the treatment of pulmonary inflammation following exposure to various Gram-negative bacteria. Peteranderl et al. expand on how alterations of ion transport function during influenza a virus (IAV) infection serve as an additional feedback loop on the respiratory inflammatory profile, that may further aggravate disease progression. They moreover discuss the recently discovered role of IFN-γ and of lung macrophage-derived TRAIL in Na,K-ATPase function and ALC. The authors critically review recent preclinical studies modulating alveolar–capillary fluid homeostasis and how these data may foster the development of novel therapeutic approaches to improve outcomes in IAV-induced lung injury. Coates et al. demonstrate how pulmonary damage inflicted by the immune response to IAV may be as important to the development of severe lung injury as the cytotoxic effects of the virus itself, especially in children. The authors highlight how activation of the NOD-like receptor protein 3 (NLRP3) inflammasome by the IAV matrix 2 (M2) proton channel and the subsequent secretion of the inflammatory cytokines IL-1β and IL-18 induce alveolar-epithelial damage and pulmonary edema under these conditions. In view of the failure of the IL1 blocking agent anakinra to improve lung injury in juvenile mice with IAV infection, the authors postulate that strategies blunting activation of NLRP3 rather than blocking certain pro-inflammatory cytokines, might be more successful to treat IAV pneumonia and IAV-associated respiratory distress, especially in children.

Patients with extensive permeability edema require ventilation strategies. However, ventilation itself may further damage the already injured lungs (4) (ventilator-induced lung injury, VILI), by augmenting inflammation and barrier dysfunction and by reducing ALC. The pro-inflammatory cytokine TNF-α, the generation of which is significantly increased in ARDS patients, plays a crucial role in the pathogenesis of VILI. TNF-α binds to two types of membrane receptors, TNF receptor 1 (TNF-R1), which carries a death domain and thus signals apoptosis, and TNF-R2, which is not a death receptor. TNF-R1 was demonstrated to mediate VILI in mice, whereas TNF-R2 rather plays a protective role (5). Using ventilated as well as spontaneously breathing acid aspiration-induced ALI mouse models, Wilson et al. investigates whether intratracheal or intranasal pretreatment of animals with a TNF-R1 (p55)-targeting domain antibody (dAb) can partially rescue the ALI phenotype. The study establishes that TNFR1-targeting dAb attenuates lung injury and edema formation in both models of acid-induced ALI, with a protection from a single dose lasting up to 24 h. Apart from its TNF receptor binding sites, TNF-α also carries a spatially distinct functional domain, which has lectin-like activity and which can be mimicked by a 17 residue peptide, the TIP peptide (a.k.a. AP301 and Solnatide) (6). The TIP peptide directly binds to the α-subunit of ENaC and as such increases both the surface expression and the open probability of the channel (7), even in the presence of bacterial toxins, such as the pore-forming toxin pneumolysin, the main virulence factor of *Streptococcus pneumoniae*. The TIP peptide in a phase 2a clinical trial in ALI patients significantly improved liquid clearance in a sub-group of patients with a SOFA score > 11 (8). Willam et al. demonstrate that the TIP peptide can activate ENaC channels displaying frameshift mutants of the α-subunit associated with pseudohypoaldosteronism type 1B (PHA-1B), a rare, life-threatening, salt-wasting disease. ENaC-α is however also a subunit of the recently discovered hybrid non-selective cation (NSC) channels in alveolar epithelial cells, together with the acid sensing ion channel 1a (ASIC-1a). Czikora et al. present original data demonstrating that apart from alveolar epithelial cells, also capillary endothelial cells express both active ENaC and NSC channels and that binding of TIP peptide to ENaC-α protects capillary barrier function in pneumolysin-treated human lung microvascular endothelial cells. These data thus indicate that the ENaC-α subunit, apart from playing a crucial role in ALC in the alveolar epithelium, can also strengthen barrier function in the capillary endothelium. Recent studies have moreover shown a protective role of this ENaC subunit, as well as of the β1 subunit of the Na-K-ATPase, in LPS-induced ALI in mice (9, 10). It is important to note that mechanisms impairing barrier function in alveolar epithelial cell monolayers can also negatively affect ENaC expression, at least partially in a transient receptor potential vanilloid 4 (TRPV4)-dependent manner (11).

Gas exchange disturbances secondary to severe pulmonary edema lead to hypoxia and hypercapnia. While O_2_ supplementation and mechanical ventilation improve hypoxia in most cases, lung protective ventilation settings (required to limit VILI) often lead to further CO_2_ retention. Vadász and Sznajder discuss how hypoxia and hypercapnia by distinct and specific molecular mechanisms impair the function of the Na,K-ATPase and ENaC, and as such blunt ALC and lead to persistence of alveolar edema. They highlight recent discoveries in sensing and signaling events initiated by hypoxia and hypercapnia, which may promote the identification of potential novel therapeutic targets in the treatment of ARDS. Gwoździńska et al. demonstrate the molecular mechanism by which elevated CO_2_ levels promote activation of inflammatory signaling pathways. These in turn facilitate phosphorylation, ubiquitination and subsequent endocytosis of ENaC-β, thereby impairing ENaC activity and ALC. Optimal gas exchange requires the integrity of the alveolar-capillary barrier and an effective ALC. As respiratory failure is a consequence of acute barrier disruption in patients with ARDS, several recent studies have focused on mechanisms that may promote both barrier repair and upregulation of ALC. Huppert and Matthay present an elegant overview demonstrating that mesenchymal stem cells (MSCs) have the capacity to both improve alveolar epithelial barrier integrity and ion channel function, including ENaC, thus improving alveolar fluid balance. As such, MSCs might represent a promising therapeutic candidate for treating ARDS.

It is important to note that while the function of the Na^+^ transporters, ENaC and Na^+^,K^+^-ATPase are critical to maintain an optimal alveolar fluid balance, the function of some other channels expressed in the distal lung epithelium and/or endothelium can foster pathological mechanisms leading to pulmonary edema. Scheraga et al. discuss how the mechano-sensitive cation channel TRPV4 affects cytokine secretion and pulmonary inflammation in asthma, cystic fibrosis, pulmonary fibrosis and ARDS. Whereas, TRPV4 alters mucociliary clearance and epithelial cell pro-inflammatory cytokine/chemokine secretion in CF, in asthma the channel mediates hypotonicity-induced airway hyper-responsiveness, but not release of Th2 cytokines. Moreover, in pulmonary fibrosis, TRPV4 mediates mechano-sensing that drives myofibroblast differentiation and experimental lung fibrosis. Recently, TRPV4 activation was demonstrated to impair ENaC-α subunit expression in alveolar epithelial cells (11). Malczyk et al. review recent data on the deleterious role of the canonical or classical transient receptor potential channel 6 (TRPC6), a Ca^2+^-permeable non-selective cation channel widely expressed in the lung and vascular tissues, in pulmonary vascular remodeling in idiopathic pulmonary arterial hypertension and in endothelial barrier disruption in ALI. Whereas, TRPC6 activators may be useful to redirect blood flow from non-ventilated regions to oxygen-rich regions of the lungs to avoid life-threatening arterial hypoxemia, TRPC6 inhibitors might represent a valuable therapeutic approach in excessive vascular remodeling or enhanced endothelial permeability.

Patients with heart failure often present with alveolar edema, primarily as a consequence of increased hydrostatic gradients secondary to elevated pulmonary vascular pressures. In contrast to severe pneumonia and ARDS, the alveolar epithelial-capillary barrier remains intact under these conditions. Azzam et al. highlight the role of the Na^+^/H^+^-exchanger (NHE) in the intracellular pH-dependent induction of pro-inflammatory cytokine generation, as can occur during acutely increased left atrial pressure or during chronic heart failure. Paradoxically, although the ability of the lungs to clear edema is impaired in acutely increased left atrial pressure, in chronic heart failure (CHF) ALC is mostly increased, particularly in compensated CHF. The authors discuss whether pro-inflammatory cytokines have a causal role in CHF pathology or whether they rather represent biomarkers for disease prognosis. They moreover critically review recent clinical trials with anti-inflammatory agents, such as the IL1 blocker anakinra in this context. Weidenfeld and Kuebler review recent data demonstrating that an acute increase in left atrial pressure (a model of acute heart failure) inhibits amiloride-sensitive Na^+^-uptake across the alveolar epithelium. They also discuss the concomitant stimulation of Na^+^- and Cl^−^-uptake via the basolaterally-expressed Na^+^-K^+^-Cl^−^ cotransporter 1 (NKCC1) and Cl^−^-secretion into the alveolar space via apically-expressed CFTR under these conditions. This may lead to Cl^−^-driven alveolar liquid secretion counteracting Na^+^-driven ALC, representing an active mechanism that drives formation of alveolar edema. In line with this notion, they demonstrate that inhibition of CFTR and NKCC1 not only blocks active alveolar liquid secretion but, via a feedback loop, also improves ALC and therefore attenuates edema formation. As such, anti-CFTR, anti-NKCC1, anti-NHE and anti-inflammatory therapies may hold promise to improve cardiogenic edema.

In summary, this Research Topic provides the reader with a combination of original and review contributions in order to present an update and an overview of the interactions between pro-inflammatory cytokines and ion transporters regulating alveolar fluid balance with relevance to pulmonary disease states, such as viral and bacterial pneumonia, ischemia-reperfusion-induced lung injury, VILI, ARDS, pulmonary hypertension and acute and chronic heart failure. Although this article series by no means addresses all aspects of this complex matter, these manuscripts may nevertheless foster the development of novel therapies toward alveolar-capillary barrier dysfunction and pulmonary edema.

## Author contributions

IV and RL edited this Research Topic and have made a substantial, direct and intellectual contribution to the work, and approved it for publication.

### Conflict of interest statement

The authors declare that the research was conducted in the absence of any commercial or financial relationships that could be construed as a potential conflict of interest.

## References

[1] Weibel ER Gomez DM. Architecture of the human lung. Use of quantitative methods establishes fundamental relations between size and number of lung structures. Science (1962) 137:577–85 14005590 10.1126/science.137.3530.577

[2] Petrache I Birukova A Ramirez SI Garcia JG Verin AD. The role of the microtubules in tumor necrosis factor-alpha-induced endothelial cell permeability. Am J Respir Cell Mol Biol. (2003) 28:574–81. 10.1165/rcmb.2002-0075OC 12707013

[3] Ware LB Matthay MA. Alveolar fluid clearance is impaired in the majority of patients with acute lung injury and the acute respiratory distress syndrome. Am J Respir Crit Care Med. (2001) 163:1376–83. 10.1164/ajrccm.163.6.2004035 11371404

[4] Goligher EC Douflé G Fan E. Update in mechanical ventilation, sedation, and outcomes 2014. Am J Respir Crit Care Med. (2015) 191:1367–73. 10.1164/rccm.201502-0346UP.Review 26075422

[5] Wilson MR Goddard ME O'Dea KP Choudhury S Takata M. Differential roles of p55 and p75 tumor necrosis factor receptors on stretch-induced pulmonary edema in mice. Am J Physiol Lung Cell Mol Physiol. (2007) 293:L60–68. 10.1152/ajplung.00284.2006 17435079

[6] Lucas R Magez S De Leys R Fransen L Scheerlinck JP Rampelberg M. Mapping the lectin-like activity of tumor necrosis factor. Science (1994) 263:814–7. 8303299 10.1126/science.8303299

[7] Czikora I Alli A Bao HF Kaftan D Sridhar S Apell HJ. A novel tumor necrosis factor-mediated mechanism of direct epithelial sodium channel activation. Am J Respir Crit Care Med. (2014) 190:522–32. 10.1164/rccm.201405-0833OC 25029038 PMC4214088

[8] Krenn K Lucas R Croizé A Boehme S Klein KU Hermann R. Inhaled AP301 for treatment of pulmonary edema in mechanically ventilated patients with acute respiratory distress syndrome: a phase IIa randomized placebo-controlled trial. Crit Care. (2017) 21:194. 10.1186/s13054-017-1795-x 28750677 PMC5531100

[9] Sternak M Bar A Adamski MG Mohaissen T Marczyk B Kieronska A. The deletion of endothelial sodium channel α (αENaC) impairs endothelium-dependent vasodilation and endothelial barrier integrity in endotoxemia *in Vivo*. Front Pharmacol. (2018) 9:178. 10.3389/fphar.2018.00178 29692722 PMC5902527

[10] Lin X Barravecchia M Kothari P Young JL Dean DA. β1-Na(+),K(+)-ATPase gene therapy upregulates tight junctions to rescue lipopolysaccharide-induced acute lung injury. Gene Ther. (2016) 23:489–99. 10.1038/gt.2016.19 26910760

[11] Dagenais A Desjardins J Shabbir W Roy A Filion D Sauvé R Berthiaume Y. Loss of barrier integrity in alveolar epithelial cells downregulates ENaC expression and activity via Ca^2+^ and TRPV4 activation. Pflugers Arch. (2018) 470:1615–31. 10.1007/s00424-018-2182-4 30088081

